# Self-organizing maps on “what-where” codes towards fully unsupervised classification

**DOI:** 10.1007/s00422-023-00963-y

**Published:** 2023-05-15

**Authors:** Luis Sa-Couto, Andreas Wichert

**Affiliations:** Department of Computer Science and Engineering, INESC-ID and Instituto Superior Técnico - University of Lisbon, Av. Prof. Dr. Aníbal Cavaco Silva, Porto Salvo, 2744-016 Lisbon Portugal

**Keywords:** Unsupervised classification, Self-organizing maps, What-Where codes, Biologically-inspired models, Visual pattern recognition

## Abstract

Interest in unsupervised learning architectures has been rising. Besides being biologically unnatural, it is costly to depend on large labeled data sets to get a well-performing classification system. Therefore, both the deep learning community and the more biologically-inspired models community have focused on proposing unsupervised techniques that can produce adequate hidden representations which can then be fed to a simpler supervised classifier. Despite great success with this approach, an ultimate dependence on a supervised model remains, which forces the number of classes to be known beforehand, and makes the system depend on labels to extract concepts. To overcome this limitation, recent work has been proposed that shows how a self-organizing map (SOM) can be used as a completely unsupervised classifier. However, to achieve success it required deep learning techniques to generate high quality embeddings. The purpose of this work is to show that we can use our previously proposed What-Where encoder in tandem with the SOM to get an end-to-end unsupervised system that is Hebbian. Such system, requires no labels to train nor does it require knowledge of which classes exist beforehand. It can be trained online and adapt to new classes that may emerge. As in the original work, we use the MNIST data set to run an experimental analysis and verify that the system achieves similar accuracies to the best ones reported thus far. Furthermore, we extend the analysis to the more difficult Fashion-MNIST problem and conclude that the system still performs.

## Introduction

Supervised deep neural networks achieved extremely high performances on image classification tasks (Goodfellow et al. 2016). However, this success depends on the difficult and expensive task of gathering labels. So, there is increased interest in unsupervised learning. Perhaps the most successful attempt at solving this issue is contrastive learning (Le-Khac et al. 2020), which leverages self-supervised learning and data augmentation to learn high quality embeddings in an unsupervised manner. Such features can then be used to train a simpler, and less data-hungry supervised model.

Meanwhile, the biologically-inspired models community has been pointing out the implausibility of supervised networks (Illing et al. 2019; Ravichandran et al. 2020; Krotov and Hopfield 2019). We can highlight the three major issues. First, these networks are learned in a non-Hebbian manner using gradient backpropagation. Second, a natural brain is able to learn numerous tasks without labels. Finally, these networks contain an output layer with one neuron per class. This grandmother cell representation forces the architecture to artificially encode the number of classes beforehand.

Several models have been recently proposed to tackle the first two issues (Illing et al. 2019; Ravichandran et al. 2020; Krotov and Hopfield 2019; Sa-Couto and Wichert 2022). More concretely, there is a focus on finding learning rules that, while staying under the generally accepted biologically-plausible umbrella, can also generate high quality hidden representations from unlabeled data. Usually, such representations are fed to a supervised single layered network for classification. Which leaves the third problem to be solved.

Recent work (Khacef et al. 2019, 2020) used self-organizing maps (SOM) to make the classifier part work without labels as well. With this approach, the neuronal structure of classification is entirely learned by a population of neurons that can have an arbitrary size. Furthermore, if new classes are introduced in the dataset, the SOM can adapt to them and so the same group of neurons may become a classifier of yet another class, thus making the model more biologically plausible.

In Khacef et al. (2019), Khacef proposed a post training labeling scheme such that the SOM’s outputs have meaning, and, with that, accuracy can be computed. So, is the system competitive with previously proposed alternatives? If the SOM receives images directly, it is not. Yet, if it receives rich hidden representations, then the answer is yes. However, to generate these representations, the authors used non-Hebbian convolutional networks as encoders (Goodfellow et al. 2016; Khacef et al. 2020), and the top performer was a supervised version.

At this point a question emerges. Is it possible to build the same type of system, with all the aforementioned advantages, but with an encoder that is not only unsupervised, but also under the biologically-plausibility umbrella? Also, can such a system remain competitive with the results achieved via deep learning-based encoders? To answer this question is the main goal of this paper.

In previous work (Sa-Couto and Wichert 2019, 2022), using visual cortex based principles we proposed the “What-Where” encoder. This network can be trained with Hebbian rules without using labels, and the embeddings it generates have been shown to work extremely well for both classification and associative memory tasks (Sa-Couto and Wichert 2020, 2021).

These reasons lead us to hypothesize that the “What-Where” encoder can precisely be the missing piece that, working in tandem with a SOM, can produce the desired end-to-end unsupervised classification system based on self-organizing maps with all the practical advantages and biological insights that it entails.

In Sect. 4, to test the hypothesis, we will follow a methodology that is similar to the aforementioned works. Concretely, we will use visual data sets like MNIST (LeCun et al. 2020) and Fashion-MNIST (Xiao et al. 2017) to test and evaluate the system. But before doing so, we will need to go through a few key steps. In Sect. 2 we will describe self-organizing maps in more detail, and show how they were used for unsupervised classification with after training labelling. After that, we will use Sect. 3 to describe how “What-Where” codes are generated and provide some intuition on why they work.

## Self-organizing maps and unsupervised classification

Self-organizing maps are a biologically inspired neural network that embeds high dimensional vectors into a grid of neurons (Kohonen 1984, 1990). In the typical format, and in the version we will consider in this work, this grid constitutes a two-dimensional square. Therefore, each neuron can be identified by its position in the grid $\textbf{p}_{n}=\left( i, j\right) \in \left[ N\right] ^{2}$ where $\left[ N\right] =\left\{ k\in \mathbb {N}:k\le N\right\}$ and *N* is the number of neurons that constitute the side of the square.

Assuming the input vectors are of dimension *d*, each neuron $\left( i,j\right)$ will have a *d*-dimensional weight vector $\textbf{w}_{i,j}$. When an input is presented to the network, all neurons compete to represent it. The unit with weights that best match the input wins and gets to define how the network is updated. More specifically, the neuron’s weights are reinforced such that the next time the same input appears it is even more likely that the same neuron wins. Furthermore, this reinforcement is applied to neighboring neurons according to a neighborhood function that depends on how far each neuron is from the winning one on the grid. This idea of neighborhood induces a topological order on the grid where neurons that are close to each other will represent similar inputs and neurons that are far away represent different ones.

At the beginning of the learning procedure, the neighborhood should be wide so that the grid can fully adapt. However, much like the learning rate, the width should decrease as the network gets more finely tuned. Both these parameters are usually denoted by $\epsilon _{t}$ and $\sigma _{t}$ respectively.

Unsupervised classification using SOMs is relatively simple. We start by applying the typical algorithm to the inputs. With that completed, we will have a grid of neurons that compete to represent each input. From each competition a winning neuron will emerge. Therefore, if the neurons are labeled such that each one corresponds to some class, then we can look at the winner as the output label. With that, we get the typical classifier (input vector in; output label out).

Although there exists no supervision in the training whatsoever, the post-training labeling is essential to be able to compute performance measures like accuracy. In Khacef et al. (2019) the authors proposed, and employed successfully, a new labeling scheme. The intuition behind it is simple. We take a relatively small subset of the data, and we label it. After that, we make the neurons compete to represent each example $\textbf{x}$ with corresponding label *y* in the subset. Then, each neuron will receive a vote to be labelled as *y* in proportion to how well it represents $\textbf{x}$. By the end of the subset, each neuron will represent the label that got more votes.

As classifiers, SOMs possess very interesting properties. Of which we can highlight two. First, no labels are really required, their only use is to compute performance indicators. The system could merely convert the winning neuron’s weights into an image and output it. There is no specific reason why a classifier must output a symbolic token representing a class.

The second key property is that these networks evade the need for grandmother cells. Therefore, no single neuron represents a whole class. Instead, the neuronal grid represents a class topology, which knows about inter-class similarities, can have an arbitrary size, and thus adapt to new classes that may emerge in the future.

## What-where codes

In this section we will provide a summarized view of the “What-Where” encoder presented in Sa-Couto and Wichert (2022). In general, one can think about it as a biologically inspired unsupervised model that generates informative representations of visual patterns. Before moving into the model equations we need to highlight two key principles that help the reader understand the flow of processing. First, an image in the retina is represented at decreasing resolution from the center (i.e. fovea) outward (Harkness and Bennet-Clark 1978). Second, vision is a process in time in which the eye performs saccadic movements across the object in sight (Liversedge and Findlay 2000).

**Fig. 1:**
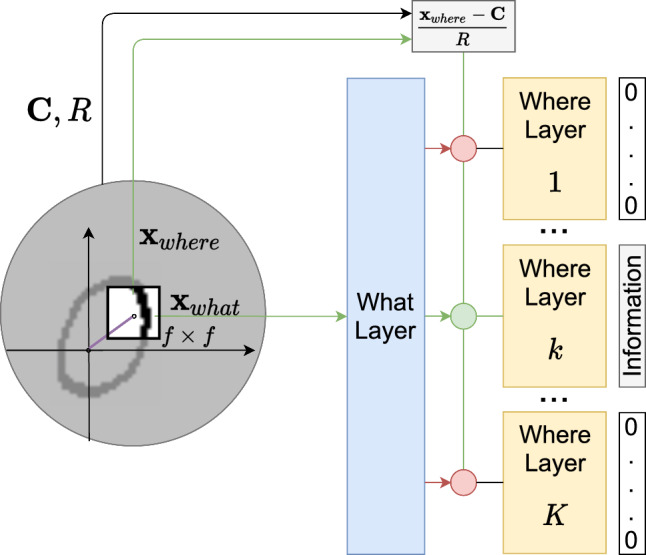
At a given instant, an $f\times f$ window $\textbf{x}_{what}$ of the image is taken as input to the “what” layer. The “what” layer routes this information to a dedicated “where” layer. There, the window’s object-dependent position $\textbf{x}_{where}$ is used to generate an encoding of the information

With that, we can define the high resolution region of our model by a window of $f\times f$ pixels. At a given time step, the model focuses on that part of the image and receives the surroundings only as information about the relative position of the current object part on the overall object. These two pieces of information, that is, the content of the window (i.e. the “what” or $\textbf{x}_{what}$) and its position (i.e. the “where” or $\textbf{x}_{where}$) are fed to a “what” layer and a set of “where” layers, respectively. Figure 1 provides a sketch of the processing at a given step. The final representation for a moment’s input will be a sparse vector that contains a description of what has been seen and where.

To understand exactly how this happens we will have to open the lid on these abstract boxes. Specifically, we will use Sect. 3.1 to do it for the “what” layer; and in Sect. 3.2 we do it for a “where” layer.

With those sections, one can grasp how the model generates an encoding vector for the object part under focus at a given instant. Yet, for classification purposes, we need a way to combine all these representations into a final, object-level encoding.

Perhaps further research into saccadic movements is needed to inspire a more sophisticated way to achieve this. However, for the purposes of this work, we do exactly what the original work did. That is, we simply do element-wise “max pooling” of the sequence of view-level encodings.

### The “what” layer

The what layer implements the winner-takes-all approach to feature mapping (Cardoso and Wichert 2010). Each of *K* units is tuned to recognize a given preferred pattern $\textbf{w}_{k},k=1,\ldots ,K$ (like a corner or an oriented line). Given an input, each unit measures a cosine similarity between its preferred pattern and that input (Sa-Couto and Wichert 2019). The usage of this measure can be viewed as applying weight normalization in a typical dot product-based layer. Such normalization is also biologically plausible since synaptic strength cannot grow unbounded (Hertz et al. 1991; Trappenberg 2009). The units then compete, and the most similar one wins firing a 1 while the others output 0. The usage of an absolute minimum threshold *T* ensures that there is not always a winner. For inputs that do not resemble any of the preferred patterns, all units will be silent. To implement this reasoning, we write the net input to unit *k* with equation 1.

1 $$\begin{aligned} net_{k}=\frac{\textbf{x}_{what}^{T}\textbf{w}_{k}}{\left\| \textbf{x}_{what}^{T}\right\| \left\| \textbf{w}_{k}\right\| } \end{aligned}$$

**Fig. 2:**
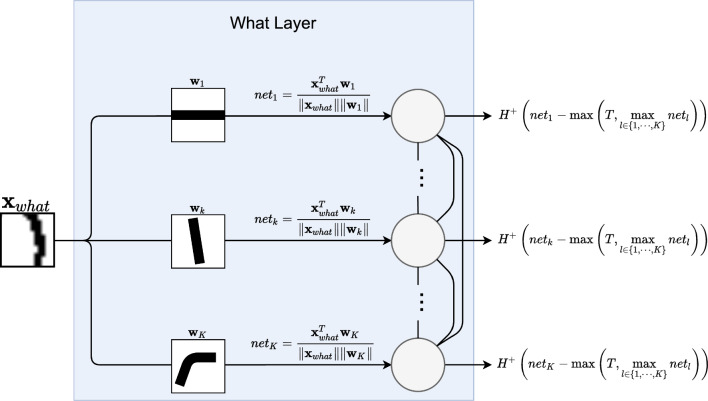
The “what” layer implements the winner-takes-all approach to feature mapping. Each of *K* units is tuned to recognize a given preferred pattern $\textbf{w}$ (like a corner or an oriented line). Given an input, each unit measures a cosine similarity between its preferred pattern and that input. The units then compete, and the most similar one wins firing a 1 while the others output 0. The usage of an absolute minimum threshold *T* ensures that there is not always a winner. For inputs that do not resemble any of the preferred patterns, all units will be silent

To define the binary activations of each unit we use the well-known, right continuous, Heaviside step activation function given in equation 2.

2 $$\begin{aligned} H\left( x\right) ={\left\{ \begin{array}{ll} 1 &{} x\ge 0\\ 0 &{} x<0 \end{array}\right. } \end{aligned}$$

Unit *k*’s output, written $what_{k}$, is the result of the competition between the layer’s units, and it can be written with equation 3.

3 $$\begin{aligned} what_{k}=H\left( net_{k}-\max \left( T,\max _{l\in \left\{ 1,\cdots ,K\right\} }net_{l}\right) \right) \end{aligned}$$

Figure 2 provides an illustration of information processing in the “what” layer.

Now that we have described the operation we are left with the learning problem: how to learn the preferred patterns $\textbf{w}_{k}$? To this end we employ the typical competitive learning approach (Rumelhart and Zipser 1985; Hertz et al. 1991; Haykin 2008) where for a given input, the winner unit gets its weights updated. One can also look at this learning approach as a variant of k-means clustering (Lloyd 1982) applied in a stochastic manner to mini-batches (Sculley 2010). All in all, we can describe the learning procedure with the rule in equation 4 where $\eta _{k}$ is the learning rate.

4 $$\begin{aligned} \textbf{w}_{k}=\textbf{w}_{k}+what_{k}\eta _{k}\left( \textbf{x}_{p}-\textbf{w}_{k}\right) \end{aligned}$$

Besides adjusting the learnable parameters, *T*, *K* and *f* play the role of hyper-parameters and have to be chosen based on the task at hand.

### The “where” layer

In general, we can look at each “where” layer as implementing a Gaussian Mixture Model (Bishop 2006; Murphy 2012) of positions in the object-dependent space. At a given time step, when processing a position, the model takes into account two pieces of information, the position itself and the context surrounding it. The context is used to change the coordinates’ system to an object-dependent one defined by a center and a radius. The center $\textbf{C}$ can be computed approximately by finding the mean position of activations whereas the radius *R* can be computed as the maximum deviation from that center (Sa-Couto and Wichert 2020). From that it follows naturally that each position is mapped to the new system through

5 $$\begin{aligned} \textbf{x}_{where}=\frac{\textbf{x}_{where}-\textbf{C}}{R}. \end{aligned}$$

This change of coordinates can be done explicitly as was just stated, or it can be done in a more biologically-plausible manner by letting the model freely saccade, and computing expected positions.

**Fig. 3:**
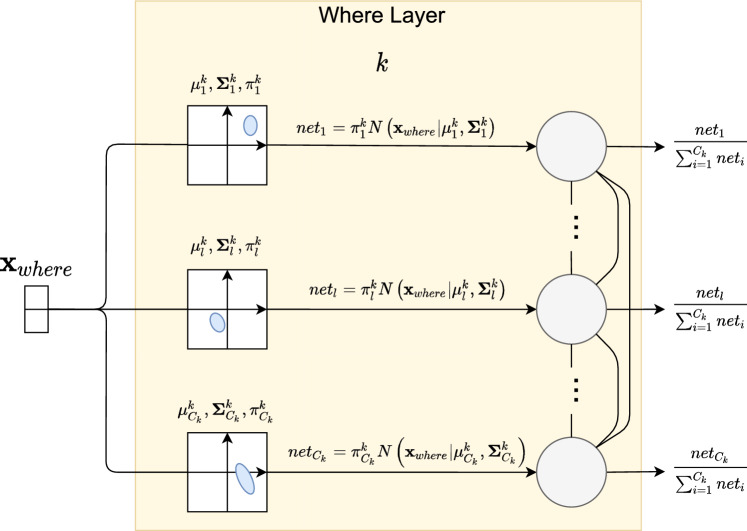
Where layer *k* has $C_{k}$ units that describe a Gaussian mixture over the space of object-dependent positions of occurrences of a given pattern. Each unit has a weight, a mean vector and a covariance matrix that together describe the area of frequently occurring positions. The final output is a soft competition to decide which component generated the observed position

Having the new coordinates, the “where” layer learns a Gaussian mixture model on this new space. To that end, each unit $l\in \left\{ 1,\ldots ,C_{k}\right\}$ in the *k*-th “where” layer is parameterized by a prior weight $\pi _{l}^{k}$, a center $\mu _{l}^{k}$ and a covariance matrix $\mathbf {\Sigma }_{l}^{k}$. The net input to a unit is the unnormalized Gaussian probability assigned by that unit to that particular position. This is expressed in equation 6.

6 $$\begin{aligned} net_{l}^{k}=\pi _{l}^{k}\mathcal {N}\left( \textbf{x}_{where}\mid {\mu }_{l}^{k},\varvec{\Sigma }_{l}^{k}\right) \end{aligned}$$

One can interpret the mean and covariance as describing a receptive field over positions.

The final output of each unit is also the product of competition between lateral units as is written in equation 7. This is basically the normalization of the probabilities using the law of total probability to get a posterior distribution.

7 $$\begin{aligned} where_{l}^{k}=\frac{net_{l}}{\sum _{i=1}^{C_{k}}net_{i}} \end{aligned}$$

With this description, we see that, since each unit represents a component, the layer’s operation is a competition to see from which component the position was generated (see Fig. 3).

In the original work (Sa-Couto and Wichert 2022), all the parameters are learned through the typical approach to learn a Gaussian Mixture. Concretely, expectation-maximization with maximum likelihood estimates (Bishop 2006). Alternatively, if one wants to increase biological plausibility, an equivalent approach is to learn the parameters online as a stochastic *k*-means clustering with the Mahalanobis distance (Melnykov and Melnykov 2014). Regardless of the approach, some architectural parameters need to be chosen beforehand. In this work, we apply the original paper’s strategy of using the Bayesian information criterion to do it (Sa-Couto and Wichert 2022).

## Experiments

As was previously described, we have good intuitive reasons to believe that using the “what-where” encoder as the first component of our system will enable us to achieve the desired goals.

However, before completely committing to that direction, we should bring other possible biologically-inspired encoders into the picture and see how they compare.

### Why use “what-where”?

To get a solid comparison ground, we chose two families of encoders. The first is one of the easiest and most well-known forms of Hebbian encoding, which is stacks of Restricted Boltzmann Machines (RBMs) (Haykin 2008). The second one is the recently proposed learning network by Krotov & Hopfield (Krotov and Hopfield 2019). Adding the “what-where” encoder, we basically have three different networks that could generate embeddings.

All three of these networks depend on choices of hyperparameters. For instance, the number of layers, the number of neurons for each layer, the learning rates and so on.

To get a sense on how the three networks compare, we performed a large random search through all three hyperparameter spaces. Every time we sampled a set of hyperparameters for one of the models, we trained it on a random sample of 2000 MNIST handwritten digits (LeCun et al. 2020). Then, we would take the trained model and use it to encode the same set of digits into the hidden space. Using these hidden representations, we would train a SOM that was also randomly hyperparameterized. With the full system trained, we would take a small, independent, labeled sample and use it to post-train label the map.

At this point, we would have a full system where accuracy could be computed. To measure it, we took an independent sample of 2000 digits. The resulting score would then be collected in the history of the corresponding encoder.

**Fig. 4:**
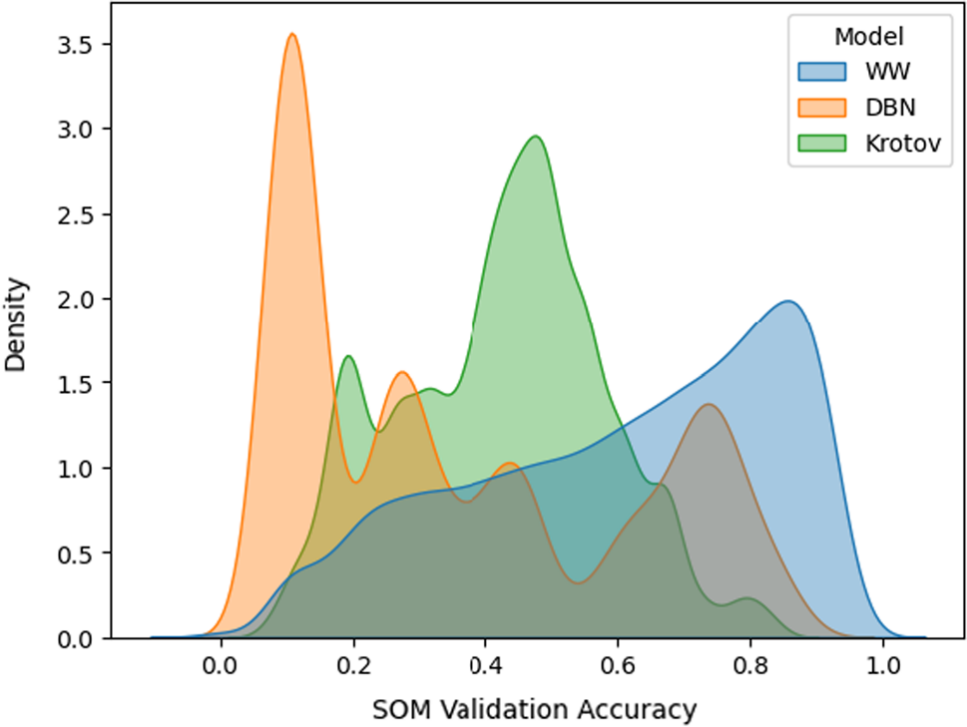
The density plots of independent validation set accuracies for each of the three systems. Comparing the three encoders, we see that stacks of RBMs have tremendous variance with the choice of parameters. On the other hand, Krotov’s method seems more stable, but just as it does not get as bad, it also does not get as good. Finally, we see that the “what-where” encoder is quite robust to parameterization and achieves the best results of the three

After repeating this search for over 1000 steps for each encoder, we collected the accuracy scores into the density plot presented in Fig. 4.

Analyzing the results, we see that the RBMs have two modes. When the hyperparameters are good, performance follows. Yet, it suffers immensely from a poorer choice of parameters. Krotov’s method is steadier as it almost never breaks down completely. However, it does not achieve the highest results. Finally, the WW encoder seems to achieve the best accuracies, and be quite consistent at that. This result is not very surprising given that it was the only one of the three to be developed specifically for visual pattern recognition tasks.

### Can the proposed system compete?

**Fig. 5:**
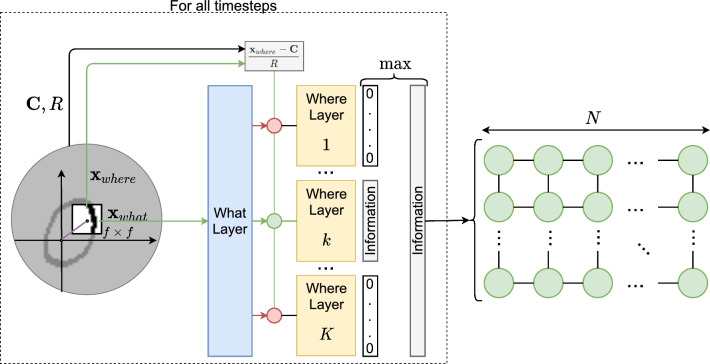
Moving the focus window through time and accumulating the outcomes by element-wise max pooling, we get a vector that encodes the whole image. That vector is then used as input to a two-dimensional $N\times N$ self-organizing map

The previous section solidified our intuition that WW would be a good candidate for the missing encoder piece in the desired target system. If we take it and connect it to a SOM, we get the end-to-end model depicted in Fig. 5.

If we recall our definition of success, we not only wanted to use a biologically-constrained encoder, but also, we wanted the system to achieve results that were competitive with the original work (Khacef et al. 2020).

There, the authors used the well-known MNIST dataset of handwritten digits (LeCun et al. 2020). Though simple, this data set is usually used as a starting benchmark for most systems, therefore we will also use it.

In theory, it could seem that two-dimensional digits are not the most natural patterns. However, biological vision has access to several 3D queues, for instance via stereopsis (Marr 1982). Therefore, only an inherently two-dimensional task is really comparable.

Having the data ready, to run experiments we need to parameterize both the encoder and the self-organizing map. If our goal was to solve the task with the maximum possible accuracy, it would make sense to list all the hyperparameters and perform an exhaustive search through them. However, as was stated before, the goal is simply to find out if the proposed system can compete.

With that in mind, we used previous literature to make informed guesses on several hyperparameters. For example, we use the exact same SOM learning rate schedule that was used in Khacef et al. (2020). Additionally, our “where” layers were automatically parameterized as is described in Sa-Couto and Wichert (2022).

The following four hyperparameters are the most task dependent, and, thus, we could not just choose them a priori.

- *K*: number of features in the “what” layer;
- *f*: size of the window;
- *T*: recognition threshold in the “what” layer;
- *N*: side of the neuron grid in the self-organizing map;

For that reason, we ran a quick random search to try to find a version of the model that could perform. Some representative results of these experiments are presented in table 1 where the accuracy on a validation set is measured.

**Table 1:** A few results of validation accuracy from a random search through hyperparameter space

T	K	f	N	Accuracy
0.7	140	5	3	0.6871
0.7	140	5	10	0.9631
0.7	140	5	15	0.9664
0.7	140	5	18	0.9731
0.7	140	5	20	0.9711
0.6	90	5	10	0.9661
0.6	190	7	25	0.9764
0.6	190	7	30	0.977
0.6	190	7	35	0.9701
0.6	190	9	10	0.9686
0.6	190	9	30	0.9701
0.6	250	9	20	0.9688
0.6	50	9	20	0.9559
0.6	190	7	20	0.9696
**0**.**6**	**190**	**7**	**30**	**0**.**9786**

One can immediately see that the system is quite robust to these variations. We can take the best performer, with parameters $\left( T=0.6,K=190,N=30\right)$ and validation accuracy of 0.9786, and compare it to the reference work.

**Fig. 6:**
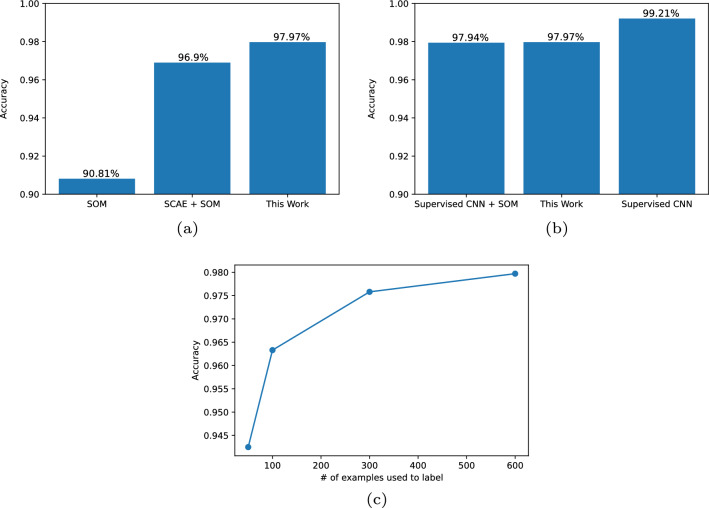
**a** A comparison between three fully unsupervised approaches. The leftmost bar presents the MNIST test set accuracy of the SOM with no feature extraction. The middle bar shows the best fully unsupervised model so far which uses sparse convolutional autoencoders as feature extractors (Khacef et al. 2020). Finally, as the legend indicates, the rightmost bar shows the results achieved by the proposed model. **b** A comparison between models that include supervised learning and the fully unsupervised one proposed in this work. On the left we present the state-of-the-art mixed model which uses a SOM on features learned through supervised learning on a convolutional network. On the right, we present a fully supervised CNN. All in all, we see that both SOM-based models are worse than the fully supervised one, however, both achieved quite respectable accuracies. Furthermore, we see that, despite being fully unsupervised, the proposed model achieves an accuracy level that is virtually equal to the mixed model’s. **c** MNIST test performance accuracy achieved by the top performer model in this work for different sizes of the labelling subset

In Khacef et al. (2020), the best fully unsupervised version of the experiments was achieved using a sparse convolutional autoencoder (SCAE) (Goodfellow et al. 2016) as an encoder. Figure 6a compares the SOM accuracy, measured on the fixed MNIST test set of 10000 digits, for the SCAE encoder, the WW encoder and no encoder at all.

Analyzing the results, we notice two key aspects. First, as expected, the SOM really does require a feature extractor to perform. Second, our biologically-inspired encoder allows the system to achieve results that are comparable to those achieved by the unsupervised deep learning technique.

As was stated before, the state-of-the-art with SOMs was achieved with a supervised encoder from a convolution network (CNN) (LeCun et al. 1998; Khacef et al. 2020). So, in Fig. 6b, we present a comparison between this mixed model, our fully unsupervised approach, and a fully supervised convolution network. We see that although the fully supervised is able to achieve a better accuracy, both of the SOM-based models are quite competitive. Furthermore, we can see that our fully unsupervised model achieves results that are comparable to those of the previously proposed mixed model.

### Are the results qualitatively interesting?

Taking the iteration of our system that was used in the previous section, we can answer two important qualitative questions.

The first one regards the size of the post-train labeling subset. Is the size of this labeled set highly impacting the scores we get? For the direct comparison that was made in the last section this is not very important given that all SOMs were labeled with the exact same amount of examples (i.e. using a subset of 1% of the MNIST training set, that is, 600 images). However, it is an important question to understand how practical such a system would be for real life usage.

In Fig. 6c we present the MNIST test accuracy for our system as the size of the labeling subset increases. Although it does play some role, the key takeaway is that with five examples per class, the model is already performing at a competitive level on this measure.

**Fig. 7:**
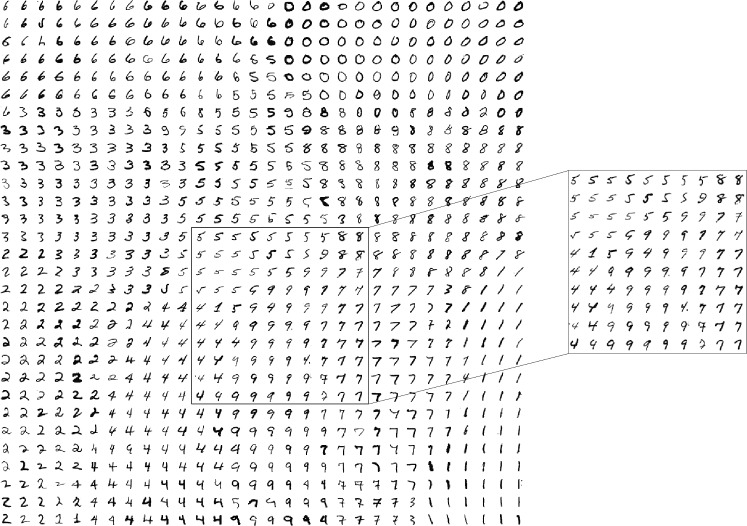
The $30\times 30$ grid of neurons can be visualized by taking handwritten digits from the dataset and assigning each to the neuron that wins the competition to represent it. By doing so, we can get a visual sense for what each neuron has learned and, thus, a sense for the network’s topology. The highlighted region illustrates a natural progression from “fours”, to “nines”, to “sevens”

Another interesting question would be to look at the class topology that was learned by the map. Figure 7 presents it. Although it is subjective to analyze, we can see that it makes sense to a human observer. Classes that are similar to us appear closer together than classes that greatly differ. For example, the highlighted region illustrates a natural progression from “fours”, to “nines”, to “sevens”.

### Can the system be used on a more difficult task?

With the advent of deep learning techniques, the machine learning community has started to regard MNIST as a relatively easy task. For that reason, the Fashion-MNIST data set was proposed as an alternative benchmark (Xiao et al. 2017).

Just like MNIST, this data set contains a training set of 60000 images, and a fixed test set of 10000. However, instead of digits, the classes are fashion items like clothing and bags.

The classes are much more difficult to separate, and the reported human performance is worse than advanced deep learning methods (Xiao et al. 2017).

Such a task is quite hard for unsupervised learning as the success of supervised deep models indicates the importance of the labels to solve the task.

However, once again, our aim is not to solve the specific task, but to prove a concept. For that reason, we decided to evaluate our system on this data set as well.

To that end, we conducted a random search through hyperparameter space, where each sample was evaluated on a small validation set. We chose the best iteration and evaluated it on the Fashion-MNIST test set. The system achieved 0.81 of accuracy. Although this value is a bit far from the accuracies achieved by end-to-end supervised CNNs with millions of parameters (in the range of 0.89 to 0.93), it is quite close to the 0.835 achieved by humans (Xiao et al. 2017).

## Conclusion

We started the work by pointing to an increased interest in unsupervised learning from the two main connectionist communities. On the one hand, the more engineering oriented deep learning community is interested in reducing the dependency on expensive labeled data. On the other hand, the biologically-constrained models community is interested in surpassing the clear implausibility of an end-to-end supervised setting in nature.

We discussed examples of unsupervised encoders that try to find rich representation spaces, such that the final supervised layers do not need so many labeled examples. Yet, in doing so, we pointed out that neither community escapes this final layer supervision which brings some important limitations besides being biologically unlikely.

Then, we described recent work where SOMs were used to tackle the final layer problem. Despite the achieved success, we pointed out that the encoders used were not Hebbian, and that the best performer was supervised.

With that in mind, we stated the main goal of the paper, which was to build a successful end-to-end unsupervised system, where the encoder was under the biologically-plausible umbrella, and the output layer was a SOM. Additionally, we defined success to be the achievement of results that were competitive with the previous approach.

At first, we explored three types of biologically-inspired encoders: stacks of restricted Boltzmann machines, networks trained with the competitive learning scheme proposed by Krotov & Hopfield, and our own “What-Where” encoder. Exploring the three approaches on the MNIST data set we concluded that the latter performed best. This result was not surprising given that, out of the three, it was the only one developed specifically for visual patterns.

We then took this version of the system and compared it with the original work and found the results extremely competitive not only when compared with the fully unsupervised version, but also with the supervised encoder version.

After that, we performed a qualitative analysis of the resulting system on two fronts. First, we noted that the accuracy measurements were robust to the number of examples used to post-train labeling of the map. Second, we looked at the neuronal topology of classes represented by the SOM and found it to be very intuitive for a human observer.

Finally, we tested the system on a much more difficult task in Fashion-MNIST. Although results are still a bit far from the best supervised models, we find that our unsupervised approach still works, achieving a performance that is not far from the reported human level.

In summary, the final system requires no labels, can be learned online using Hebbian rules, and does not depend on an output layer of grandmother cells. This last property allows it to, in principle, adapt to new classes that may appear in the data without having to retrain or change the architecture. Such a model shares several characteristics with the flexible vision we see in biology and is thus a very interesting candidate to be part of a learning system that is inspired by knowledge of the brain.
